# Supplementary material for: Niclosamide and Pyrvinium Are Both Potential Therapeutics for Osteosarcoma, Inhibiting Wnt–Axin2–Snail Cascade
Source: Cancers (Basel). 2021 Sep 15;13(18):4630. doi: 10.3390/cancers13184630 (PMC8464802; doi:10.3390/cancers13184630)
Supplement: Supplementary file 1 [file cancers-13-04630-s001.zip › cancers-1375202-supplementary.pdf]

Figure S1. Uncropped images of all the Western Blot data

Fig. 1C

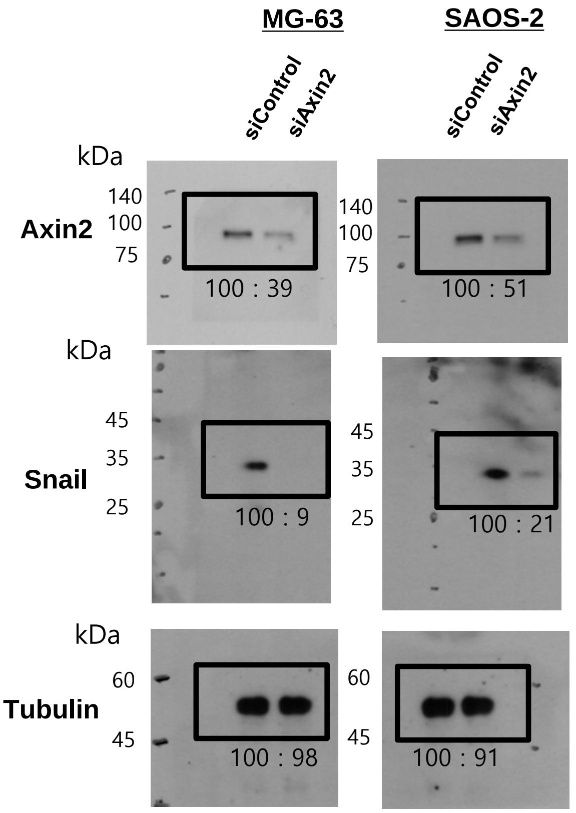

Fig. 3B

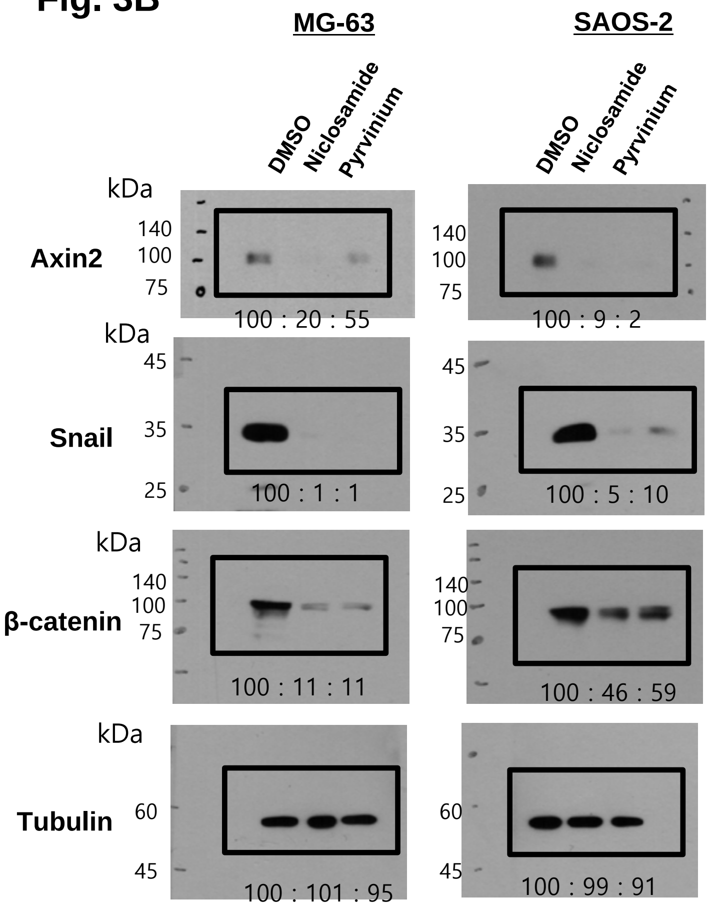

Fig. 4A

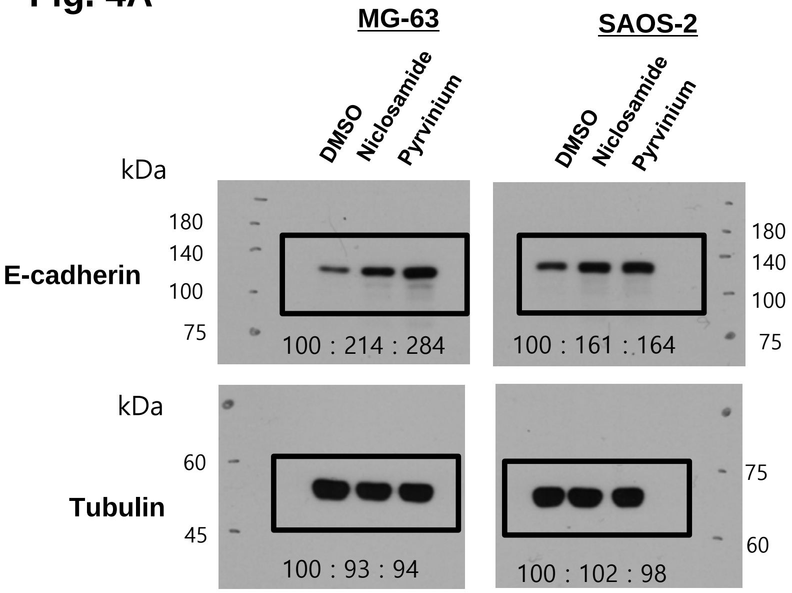

Table S1. Primer sequences used for real-time quantitative PCR

Gene	Forward Sequence(5'~3')	Reverse Sequence(5'~3')
AXIN2	AAGGGCCAGGTCACCAAAC	CCCCCAACCCATCTTCGT
Snail	TCTCTGAGGCCAAGGATCTC	CTTCGGATGTGCATCTTGAG
CD44	CCTCTTGGCCTTGGCTTTG	TCCATTGCCACTGTTGATCAC
EPHB2	CAGCCCAATGGCGTGATC	TTTATGGCTGTGGCGTTGTACT
EPHB3	CGCGAGTCAAGCCAGAACA	TGCAGTCACGCACAGTGAACT
FGF18	CTCTACAGCCGGACCAGTG	CCGAAGGTGTCTGTCTCCAC
ID2	ACCAGAAGGATCCAGTATTCAGTC	CAGTAGGCTTGTGTCAAAAAGGTA
MYCN	GACCACGTGCCGGAGTTG	GAGTGGACATACTCAGTGGCCTTT
NOTUM	AAACATGGTCTTCATCCCCTACT	ATGAAGGCGTACTCGTTCTTCTC
TCF4	CAGCAAGCACTGCCGACTAC	GGAGCTAGGGAAAGTGCTGGTT
TCF7	CCCCAACTCTCTCTCTACGAACA	TGAACTTGCTTCTGGCTGATGT
Occludin	CGGTCTAGGACGCAGCAGAT	AAGAGGCCTGGATGACATGG
E-cadherin	TGAGTGTCCCCCGGTATCCTC	CAGTATCAGCCGCTTTCAGATTTT
Fibronectin	CAGGATCACTTACGGAGAAACAG	GCCAGTGACAGCATACACAGTG
Vimentin	AATGGCTCGTCA CCTTCGTGAAT	CAGATTAGTTTCCCTCAGGTTCAG
GAPDH	TCCGCGGCTATATGAAAACAG	TCGTAGTGGGCTTGCTGAA
